# Anticancer Activity of Gukulenin A Isolated from the Marine Sponge *Phorbas gukhulensis* In Vitro and In Vivo

**DOI:** 10.3390/md17020126

**Published:** 2019-02-21

**Authors:** Ji-Hye Ahn, Jeong-Hwa Woo, Jung-Rae Rho, Jung-Hye Choi

**Affiliations:** 1 College of Pharmacy, Kyung Hee University, Seoul 02447, Korea; jihyeahn21@gmail.com (J.-H.A.); jhwoo@khu.ac.kr (J.-H.W.); 2 Department of Life and Nanopharmaceutical Sciences, Kyung Hee University, Seoul 02447, Korea; 3 Department of Biochemistry and Molecular Genetics, University of Virginia School of Medicine, Pinn Hall 1232, Charlottesville, VA 22908, USA; 4 Department of Oceanography, Kunsan National University, Jeonbuk 54150, Korea; jrrho@kunsan.ac.kr

**Keywords:** gukulenin A, *Phorbas gukhulensis*, ovarian cancer, apoptosis, tumor xenograft model

## Abstract

Gukulenin A is a bis-tropolone tetraterpenoid isolated from the marine sponge *Phorbas gukhulensis*. In this study, we examined the anticancer activities of gukulenin A in ovarian cancer cell lines (A2780, SKOV3, OVCAR-3, and TOV-21G) and in an ovarian cancer mouse model generated by injecting A2780 cells. We found that gukulenin A suppressed tumor growth in A2780-bearing mice. Gukulenin A markedly inhibited cell viability in four ovarian cancer cell lines, including the A2780 cell line. Gukulenin A treatment increased the fraction of cells accumulated at the sub G1 phase in a dose-dependent manner and the population of annexin V-positive cells, suggesting that gukulenin A induces apoptotic cell death in ovarian cancer cells. In addition, gukulenin A triggered the activation of caspase-3, -8, and -9, and caspase inhibitors attenuated gukulenin A-induced A2780 cell death. The results suggest that gukulenin A may be a potential therapeutic agent for ovarian cancer.

## 1. Introduction

Worldwide, ovarian cancer is the most lethal gynecological malignancy. Approximately 204,000 women are diagnosed with ovarian cancer, and among them, approximately 140,200 women die of this cancer each year [1]. Despite improvements in the standard management for ovarian cancer, the long-term survival rate of patients still remains poor [2]. Therefore, it is urgent to discover novel therapeutic agents that improve survival rate and quality of life for patients with ovarian cancer.

Marine sponges (Porifera) have been recognized as the most prolific marine sources of secondary metabolites with diverse biological activities, including immunosuppressive, neuroprotective, neurosuppressive, antibacterial, anti-inflammatory, antifungal, antiviral, and anticancer activities [3,4]. In a previous study, we isolated two novel bis-tropolone tetraterpenoids, gukulenin A and B, from the marine sponge, *Phorbas gukhulensis*, collected off the coast of Gageodo, Korea and examined their cytotoxicity in various cancer cells [5]. Compared to gukulenin B, gukulenin A showed a much higher cytotoxicity in pharynx cancer FaDu cells, colon cancer HCT-116 cells, renal cancer SN12C cells, and stomach cancer MKN45 cells with observed IC_50_ values of 57, 62, 92, and 130 nM, respectively. A recent report also showed that gukulenin A markedly inhibited cell viability in lung cancer A549 cells and leukemia K562 cells with IC_50_ values of 0.32 μM and 0.26 μM, respectively [6]. Although the cytotoxic activity of gukulenin A in cancer cells has been suggested, the anticancer effects of gukulenin A and its underlying molecular mechanism, to our knowledge, have never been reported.

The aim of this study was to examine the anticancer activities of gukulenin A in ovarian cancer cell lines and in a xenograft mouse model of ovarian cancer. We observed that gukulenin A inhibited tumor growth in vivo and induced apoptotic cell death via the activation of caspases in ovarian cancer cells in vitro.

## 2. Results

### 2.1. Gukulenin A Inhibited Tumor Growth in an Ovarian Cancer Xenograft Mouse Model

**Figure 1:**
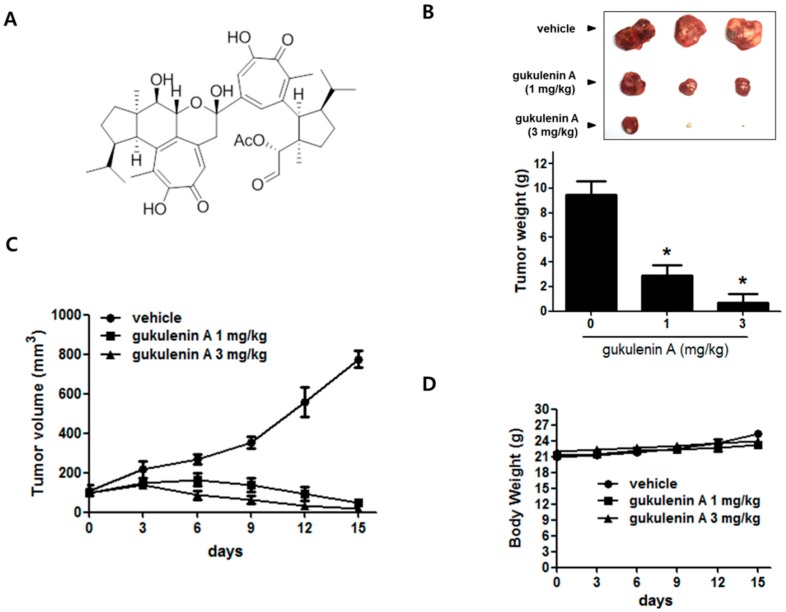
Effect of gukulenin A on tumor growth in a mouse xenograft model. (**A**) Chemical structure of gukulenin A isolated from the marine sponge *Phorbas gukhulensis*. (**B–D**) A2780 xenografts were treated with vehicle or gukulenin A (1 and 3 mg/kg) every three days for 15 days. (**B**) At day 15, the mice were sacrificed and tumor weights (g) were measured. Tumor volume (mm^3^) (**C**) and body weight (**D**) changes were measured every three days for 15 days. The data are expressed as means ± SD (*n* = 5). * *p* < 0.05 as compared with the vehicle-treated group.

In our ongoing project to search for a novel marine-derived anticancer compound, gukulenin A exhibited significant cytotoxic activity in a preliminary in vitro screening against human ovarian cancer A2780 cells. Thus, we investigated the effects of gukulenin A (Figure 1A) on tumor growth in an ovarian cancer xenograft mouse model. Gukulenin A treatment (1 and 3 mg/kg) showed a higher tumor growth suppression than vehicle treatment (Figure 1B,C). The administration of gukulenin A at 1 and 3 mg/kg doses resulted in 69.30% and 92.43% tumor weight inhibition, respectively (Figure 1B). In addition, the mean tumor volume in the vehicle-treated group increased from 100 mm^3^ to 774.65 ± 72.97 mm^3^, whereas the mean tumor volume considerably decreased from 100 mm^3^ to 50.15 ± 18.78 mm^3^ and 17.67 ± 22.24 mm^3^ in 1 and 3 mg/kg gukulenin A treated-mice, respectively (Figure 1C). However, a considerable decrease in the body weights of the gukulenin A-treated mice was not observed (Figure 1D).

### 2.2. Gukulenin A Decreased Human Ovarian Cancer Cell Viability

**Figure 2:**
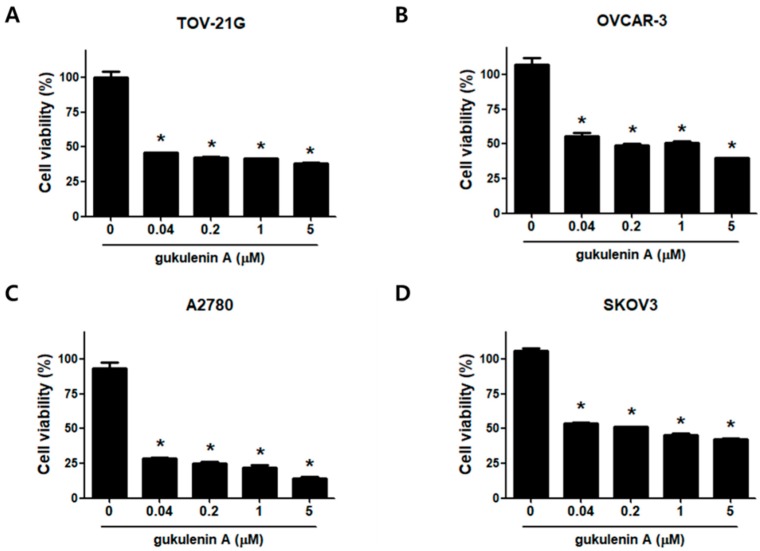
Effect of gukulenin A on cell viability in human ovarian cancer cells. TOV-21G (**A**), OVCAR-3 (**B**), A2780 (**C**), and SKOV3 (**D**), were treated with the indicated concentration (0.04, 0.2, 1, and 5 μM) of gukulenin A for 48 h. The effect of gukulenin A on cell viability was determined by MTT assay. Results are the combined data (mean ± SD) from three independent experiments. * *p* < 0.05 as compared with the untreated group.

Considering our promising in vivo results, we further investigated how gukulenin A inhibits tumor growth using ovarian cancer cell lines. First, an MTT (3-(4,5-Dimethylthiazol-2-yl)- 2,5-diphenyl-tetrazolium bromide) assay was performed to examine the effects of gukulenin A on the viability of human ovarian cancer cells TOV-21G, OVCAR-3, A2780, and SKOV3. Treatment with gukulenin A considerably inhibited cell viability in all four ovarian cancer cells in a dose-dependent manner (Figure 2). Among the four cell lines tested, A2780 cells were the most sensitive to gukulenin A treatment; therefore, subsequent experiments were conducted using the A2780 cells.

### 2.3. Gukulenin A-Induced Apoptotic Cell Death in Human Ovarian Cancer Cells

**Figure 3:**
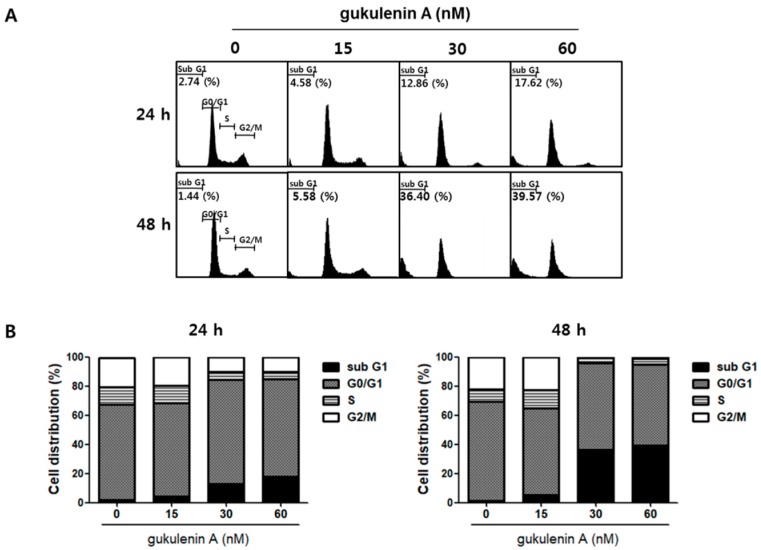
Effects of gukulenin A on cell-cycle regulation in human ovarian cancer cells. A2780 cells were treated with the indicated concentration of gukulenin A (15, 30, and 60 nM) for 24 and 48 h, and then stained with propidium iodide (PI). (**A**) Flow cytometry analysis was performed for the cell-cycle distribution profiles of the cells. (**B**) The percentages of cells in the sub G1, G0/G1, S, and G2/M phases of the cell cycle were shown as a graph. The data are representative of three independent experiments.

**Figure 4:**
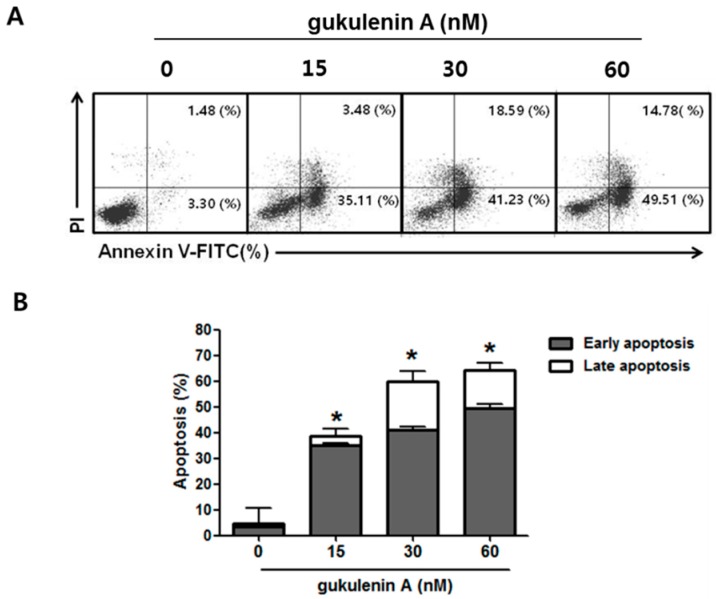
Effect of gukulenin A on the induction of apoptosis in human ovarian cancer cells. A2780 cells were treated with the indicated concentration of gukulenin A (15, 30, and 60 nM) for 48 h and then double stained with PI and Annexin V-FITC. (**A**) Flow cytometry analysis was performed for the staining profiles of the cells. The data are representative of three independent experiments. (**B**) The respective cell percentages in early and late apoptosis are presented in the bar graph. The values shown are the mean of three independent experiments. * *p* < 0.05 as compared with the untreated group.

To further determine whether the inhibitory effect of gukulenin A on cancer cell viability was induced by cell cycle arrest, cell cycle distribution was analyzed in A2780 cells following gukulenin A treatment. As shown in Figure 3, gukulenin A induced an increase in the sub G1 phase population of A2780 cells; however, it failed to induce cell cycle arrest. After treatment with 15, 30, and 60 nM of gukulenin A for 24 and 48 h, the percentage of sub G1 phase cells was 4.58%, 12.86%, and 17.62% at 24 h and 5.58%, 36.40%, and 39.57% at 48 h, respectively. These data suggest that the inhibitory effects of gukulenin A on cell viability was mediated by the induction of cell death rather than cell cycle arrest. We further investigated whether gukulenin A-induced cell death was associated with the induction of apoptosis using Annexin V-FITC and PI double staining assays. Gukulenin A increased the proportion of early (Annexin V+/PI-, lower right) and late apoptotic (Annexin V+/PI+, upper right) cells in a dose-dependent manner (Figure 4A,B). These results suggest that gukulenin A induced the cell death of human ovarian cancer cells by the induction of apoptosis.

### 2.4. Caspases Are Involved in Gukulenin A-Induced Apoptosis in Human Ovarian Cancer Cells

**Figure 5:**
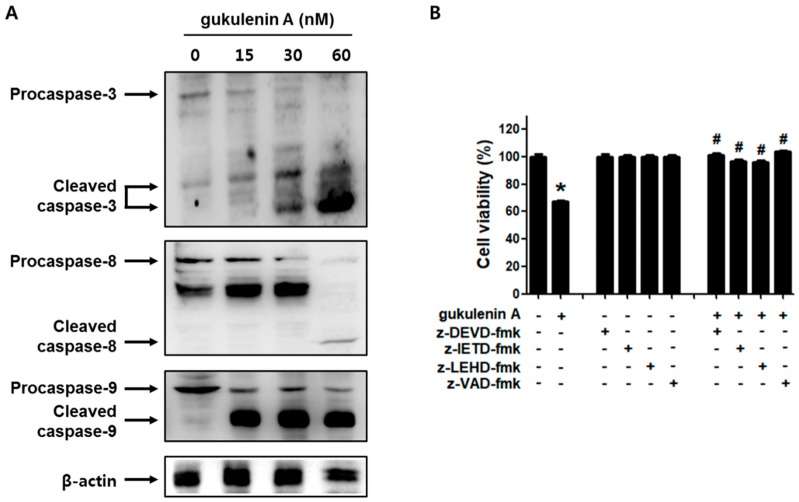
Involvement of caspases in gukulenin A-induced apoptosis in human ovarian cancer cells. (**A**) The effect of gukulenin A on caspase activation in human ovarian cancer cells. After 48h treatment of A2780 cells with gukulenin A (15, 30, and 60 nM), Western blot assay was performed to determine the levels of pro/cleaved caspase-3, -8, and -9. β-Actin was used as an internal control. The immunoblots are representative of three independent experiments. (**B**) The effect of caspase inhibitors on gukulenin A-induced cell death in human ovarian cancer cells. A2780 cells were treated with gukulenin A (60 nM) in the presence of caspase-3 inhibitor z-DEVD-fmk (50 μM), caspase-8 inhibitor z-IETD-fmk (50 μM), caspase-9 inhibitor z-LEHD-fmk (50 μM), and broad caspase inhibitor z-VAD-fmk (50 μM) for 24 h. An MTT assay was performed to assess the cell death. * *p* < 0.05 as compared with the untreated group. # *p* < 0.05 as compared with the gukulenin A only treated group.

To determine whether the caspases were involved in gukulenin A-induced apoptosis in human ovarian cancer cells, the activation of caspase-3, -8, and -9 was evaluated after treatment with gukulenin A. Western blot analysis showed that gukulenin A treatment increased the levels of the cleaved forms of caspase-3, -8, and -9 in A2780 cells (Figure 5A). We confirmed the involvement of the caspases in gukulenin A-induced apoptosis using specific caspase inhibitors. As shown in Figure 5B, z-DEVD-fmk, z-IEVD-fmk, z-LEHD-fmk, and z-VAD-fmk considerably negated the cell death caused by gukulenin A treatment in A2780 cells. These results suggest that gukulenin A induces apoptosis through the caspase pathway in human ovarian cancer cells.

## 3. Discussion

Marine secondary metabolites, whose biochemical structural diversity is yet unexploited, have been regarded as a promising source of novel anticancer drugs [7]. A significant number of marine natural products are currently in preclinical or early clinical development, and a few marine natural products, such as trabectedin (ET-743) [8,9] and eribulin mesylate (E7389) [10] have been approved for cancer treatment. Marine sponges, of all marine organisms, are the richest source of natural marine products, attributing to 30% of all natural marine products isolated to date [11]. Particular alkaloids, sterols, nucleosides, peroxides, terpenes, fatty acids, alkaloids, and peptides from marine sponges have been suggested to have biological activities and many of them show anticancer potential [12]. Cytarabine (cytosine arbinoside, ara-C) and eribulin mesylate (E7389), which were originally isolated from marine sponges, have already been approved for the treatment of leukemia and liposarcoma, respectively. Cytarabine is a synthetic analog of nucleoside spongothymidine extracted from the Caribbean sponge *Tectitethya crypta* [13] and eribulin mesylate is a synthetic compound derived from polyketide halichondrin B, which is isolated from the marine sponge *Halichondria okadai* [14].

As per our knowledge to date, there is no approved marine sponge product for the treatment of ovarian cancer, although several reports have shown the potential anticancer effects of marine sponge products in ovarian cancer cells. For example, discodermolide, isolated from *Discodermia dissolute*, induces microtubule-stabilization and inhibits the growth of paclitaxel-resistant ovarian cancer cells [15]. Microcionamides A, C, and D (1–3), cyclic cysteine-bridged peptides isolated from *Clathria basilana*, showed cytotoxicity in A2780 ovarian cancer cells with IC_50_ values ranging from 0.45 μM to 28 μM [16]. Holland et al. isolated four new trihydroxysterols from the Australian sponge *Psammoclema* sp. and reported their cytotoxic activity in A2780 cells with IC_50_ values ranging from 5.3 μM to 19 μM [17]. Leiodelide A, isolated from the deep-water marine sponge *Leiodermatium*, also showed cytotoxic activity (IC_50_ = 0.25 μM) in OVCAR-3 cells. For most of the isolated marine sponge compounds, the reported activities have been observed in vitro using ovarian cancer cells. We found only one reported study to have investigated the effect of marine sponge product on an ovarian cancer animal model [18]. In that study, discodermolide isolated from the Caribbean marine sponge *Discodermia dissolute* enhanced the tumoricidal effect of taxol; however, discodermolide treatment alone did not show any effect on tumor growth in SKOV3-bearing xenograft mice [18]. In the present study, we demonstrated that gukulenin A, a metabolite of the Korean marine sponge *Phorbas gukhulensis*, markedly inhibited tumor growth in xenograft mice with ovarian cancer without any considerable adverse effect on their body weights. To the best of our knowledge, this is the first report that a marine sponge product alone can inhibit the tumor growth in an in vivo model of ovarian cancer. We further demonstrated that gukulenin A has a potent cytotoxic activity in various ovarian cancer cells with IC_50_ values ranging from 0.03 μM to 0.36 μM (Table S1). It is of note that the cytotoxic activity of gukulenin A is more potent than that of cisplatin in all ovarian cancer cells tested in this study. Cancer treatment failure is often due to primary or acquired resistance to the chemotherapeutic agents. Although platinum/taxane combination therapy is still some of the most effective and commonly used for the treatment of ovarian cancer, acquired drug resistance has become a major obstacle in managing most ovarian cancer patients. Thus, current efforts are being directed toward discovering novel approaches to overcome the drug resistance. In this regard, it should be further investigated whether gukulenin A can overcome the chemoresistance in ovarian cancer cells.

In addition to gukulenin A, several novel compounds have been isolated from *Phorbas* sp. and many of them have shown cytotoxic activities in some human cancer cells. For example, gagunin H, a new diterpene acid of a bisabolane-related skeletal class, exhibited moderate cytotoxicity in K562 human erythroleukemia cells [19]. Three new sesterterpenoids, phorbaketals A, B, and C, which have a spiroketal of the hydrobenzopyran moiety, showed cytotoxic activities against human hepatoma, lung cancer, and colorectal cancer cell lines [20]. Recently, four new diterpenoid pseudodimers, designated gukulenins C–F, were isolated from *Phorbas gukhulensis* along with gukulenin A and showed considerable cytotoxic activities against human erythroleukemia and lung cancer cells with IC_50_ values ranging from 0.04 μM to 0.55 μM [6]. To date, the effects of those compounds isolated from *Phorbas* sp. on ovarian cancer cells remain to be further studied.

Apoptosis is the best studied mode of programed cell death to eliminate aged or damaged cells from the body. Cancer cells lose their ability to go through apoptosis-induced cell death leading to uncontrolled cell growth, resulting in tumor growth, chemoresistance, and recurrence of cancer. Thus, triggering the apoptosis of cancer cells has been considered as a strategy to treat cancer. In fact, many conventional anticancer therapies are shown to induce tumor cell death through the apoptotic pathways. In this study, we demonstrated that gukulenin A induced apoptotic cell death via the activation of caspases in human ovarian cancer cells. In addition to gukulenin A, many compounds obtained from sea sponges showed their potential anticancer activity through the stimulation of apoptosis [21]. The dibromotyrosine derivative (1′R,5′S,6′S)-2-(3′,5′-dibromo-1′,6′- dihydroxy-4′-oxocyclohex-2′-enyl) acetonitrile, isolated from *Pseudoceratina* sp., exhibited apoptotic activity by the activation of caspase-9 in leukemia K562 cells [22]. Candidaspongiolide, a novel polyketide from *Candidaspongia* sp., promoted apoptosis in human U251 glioma and HCT116 colon carcinoma cells by triggering caspase-12 and caspase-3 activation [23].

Trabectedin (ET-743, Yondelis^®^), a tetrahydroisoquinoline alkaloid originally isolated from the marine tunicate *Ecteinascidia turbinate*, was the first marine anticancer agent approved by the European Union for the treatment of advanced soft tissue sarcoma. Notably, it has been evaluated in a Phase III trial for the treatment of refractory/relapsed multiple ovarian cancer [8,9]. It is of interest that the anticancer activities of trabectedin have been demonstrated to be associated with the modulation of tumor microenvironment as well as the inhibition of tumor cell growth [24]. The tumor microenvironment is a heterogeneous population of cells consisting of the cancer cells and supporting non-cancer cells such as tumor-associated macrophages (TAM). It has previously been shown that these supporting cells are recruited by cancer cells and promote proliferation, angiogenesis, and metastasis [25,26,27]. In this regard, novel and more effective anticancer therapies targeting not only the tumor but also its microenvironment have been investigated. Trabectedin has been reported to reduce the production of MCP-1 and RANTES, required for the recruitment of TAMs and VEGF needed for tumor angiogenesis in cancer cells [28]. We found that gukulenin A markedly inhibited the expression of MCP-1, RANTES, and VEGF in the xenograft ovarian tumor mouse model (Data not shown). These data suggest that the anticancer activity of gukulenin A is possibly associated with the modulation of the tumor microenvironment as well as the induction of apoptotic cancer cell death; however, further research is needed to evaluate this.

As per our knowledge, this is the first study to demonstrate the anticancer activity of gukulenin A in an in vitro and in vivo model of ovarian cancer. Gukulenin A suppressed ovarian tumor growth in a xenograft mouse model and induced caspase-dependent apoptosis in human ovarian cancer cells. These findings suggest that gukulenin A, a bis-tropolone tetraterpenoid isolated from the marine sponge *Phorbas gukhulensis*, is a promising therapeutic agent for patients with ovarian cancer.

## 4. Materials and Methods

### 4.1. Isolation of Gukulenin A

As previously reported [5], gukulenin A (>95% purity; Figure S1) was isolated from the marine sponge *Phorbas gukhulensis* collected off the coast of Gageodo, Korea. Briefly, the MeOH extract of the sponge was partitioned between CH_2_Cl_2_ and H_2_O. The organic layer was once more partitioned between 15% aqueous MeOH and n-hexane, and then the polar layer was subjected to reversed-phase flash column chromatography to yield seven subfractions. The subfraction showing activity was chromatographed on reversed-phase HPLC to isolate gukulenin A. Gukulenin A was determined as a bis-tropolone tetraterpenoid by a combination of the spectral and chemical methods.

### 4.2. Materials

Fetal bovine serum (FBS), Roswell Park Memorial Institute (RPMI) 1640 medium, streptomycin sulfate, and penicillin were purchased from Life Technologies Inc. (Grand Island, NY, USA). MTT was acquired from Molecular Probes Inc. (Eugene, OR, USA). Annexin V-fluorescein isothiocyanate (FITC) and phenylmethylsulfonylfluoride (PMSF) were obtained from BD Biosciences (San Jose, CA, USA). Propidium iodide (PI) and 2-mercaptoethanol were purchased from Sigma Chemical (St. Louis, MO, USA). Caspase-3 inhibitor z-DEVD-fmk, caspase-8 inhibitor z-IETD-fmk, caspase-9 inhibitor z-LEHD-fmk, and broad caspase inhibitor z-VAD-fmk were from Calbiochem (Bad Soden, Germany). The ECL (enhanced chemiluminescence) reaction kit was purchased from Amersham Pharmacia Biotech (Piscataway, NJ, USA).

### 4.3. Cell Culture

A2780, SKOV3, OVCAR-3, and TOV-21G human ovarian cancer cell lines were purchased from American Type Culture Collection (ATCC; Manassas, VA, USA). RPMI 1640 medium containing 5% FBS, streptomycin sulfate (100 μg/mL), and penicillin (100 U/mL) were used for the cell culture. The cells were incubated at 37 °C in a 5% CO_2_ and 95% air humidified atmosphere.

### 4.4. Animal Study

BALB/c athymic female nude mice (*n* = 15) weighing 20–23 g were purchased from NARA Biotech (Seoul, Korea). A2780 cells (1 × 10^7^ cells) were subcutaneously injected into the flank of each mouse. The mice were randomly divided into 3 groups (5 mice/group) when the average tumor volume reached 100 mm^3^, and injected intratumorally with gukulenin A (1 and 3 mg/kg) once every three days for 15 days. At day 15, the mice were sacrificed and tumor weights were measured. The tumor volume was calculated using the formula: tumor volume (mm^3^) = 1/2 (L ×W^2^) (L: tumor length and W: tumor width). Tumor volume (mm^3^) and body weight changes were measured every three days for 15 days. Animal treatment and maintenance were conducted in accordance with protocols (KHUASP(SE)-18-037) approved by the Institutional Animal Care and Use Committee of Kyung Hee University.

### 4.5. Cell Viability

Cell viability was evaluated using an MTT assay. Briefly, human ovarian cancer cells were seeded at a density of 9 × 10^4^ cells/mL in each well of a 96-well plate and incubated for 24 h. Gukulenin A dissolved in DMSO (dimethyl sulfoxide) was diluted with medium, added on to each well, and incubated for 48 h. Following 4 h incubation with MTT solution (final concentration: 0.5 mg/mL), the purple formazan crystals formed were dissolved with DMSO. The optical density was assessed at 540 nm by a microplate spectrophotometer (Spectra Max; Molecular Devices, Sunnyvale, CA, USA).

### 4.6. Flow Cytometry Analysis

For cell cycle analysis, the cells were incubated with 70% ice-cold EtOH at 4 °C for 1 h after washing with ice-cold phosphate buffered saline (PBS). The cells were suspended in a PI solution (50 μg/mL) containing RNase A (250 μg/mL). After 30 min incubation in a dark place, the mixture was analyzed by Guava^®^ easyCyte flow cytometers (Millipore; Billerica, MA, USA). For apoptosis analysis, Annexin V and PI double staining was performed. The cells were incubated with staining solution containing 5 μL of PI (50 μg/mL), 5 μL of FITC-conjugated Annexin V, and 100 μL of binding buffer (140 mM NaCl, 2.5 mM CaCl_2_, 10 mM HEPES/NaOH, pH 7.4) for 15 min in a dark place. Guava^®^ easyCyte system (Millipore; Billerica, MA, USA) was used for the flow cytometry analysis.

### 4.7. Western Blot Analysis

Protein lysis buffer (Intron Biotechnology, Seoul, South Korea) was used for the protein extraction from A2780 cells. Protein concentrations were measured by the Bradford assay. The protein samples were mixed with SDS-PAGE sample buffer and heated for 5 min at 95 °C. The samples were loaded on a gel for SDS-PAGE. Following electrophoretic separation, separated proteins were blotted to PVDF (polyvinylidene difluoride) membranes. After blocking with 2% bovine serum albumin (BSA) for 30 min, the membranes were incubated overnight at 4 °C with diluted primary antibodies against caspase-3, -8, 9, and β-actin in TBS-T (Tris-buffered saline containing Tween-20) with 2% BSA. After a subsequent washing with TBS-T, the membranes were incubated with an appropriate secondary antibody at room temperature for 2 h. Immunoreactive bands were visualized by the ECL kit and analyzed by ImageQuant Las-4000 (GE Healthcare Life Science, WI, USA). Caspase-3 and β-actin antibodies were acquired from Santa Cruz Biotechnology (Santa Cruz, CA, USA). Caspase-8 antibody was obtained from BD Biosciences. Caspase-9 antibody was purchased from Cell Signaling (Beverly, MA, USA).

### 4.8. Statistical Analysis

The data were presented as mean ± standard deviation (SD). Student’s t-test was performed to compare two groups. For a comparison of more than two groups, one-way ANOVA was used. Under all circumstances, *p* < 0.05 was considered to be significant.

## Supplementary Materials

The following are available online at https://www.mdpi.com/1660-3397/17/2/126/s1, Table S1: Cytotoxic activity of gukulenin A in human ovarian cancer cells, Figure S1: H-NMR spectrum of gukulenin A.
