# Supplementary material for: Anticancer Activity of Gukulenin A Isolated from the Marine Sponge Phorbas gukhulensis In Vitro and In Vivo
Source: Mar Drugs. 2019 Feb 21;17(2):126. doi: 10.3390/md17020126 (PMC6410303; doi:10.3390/md17020126)
Supplement: Supplementary file 1 [file marinedrugs-17-00126-s001.pdf]

Supplementary Materials:

Table S1. Cytotoxic activity of gukulenin A in human ovarian cancer cells.

cell lines	IC_{50}[a] (μM)	
	**gukulenin A**	**cisplatin**
TOVG-21G	0.04 ± 0.00	42.67 ± 5.25
OVCAR-3	0.13 ± 0.02	11.76 ± 2.17
A2780	0.03 ± 0.00	20.61 ± 1.89
SKOV3	0.36 ± 0.06	28.90 ± 1.07

[a]IC50 is defined as the concentration that reduces the number of cells by 50% compared with control cultures. The values represent the means of the results from three independent experiments with similar patterns.

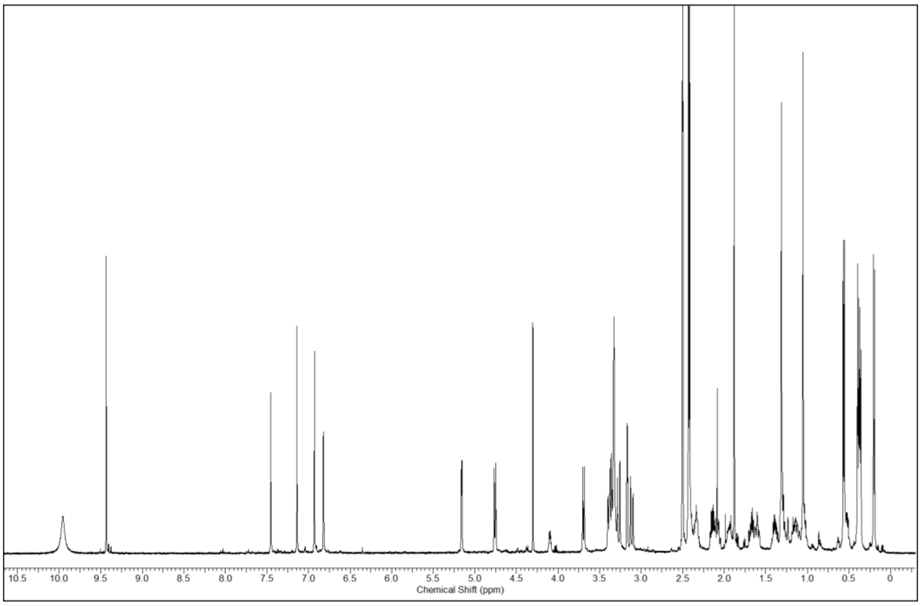

Figure S1. H-NMR spectrum of gukulenin A.
